# Peripheral reductive capacity is associated with cognitive performance and survival in Alzheimer's disease

**DOI:** 10.1186/1742-2094-3-4

**Published:** 2006-03-03

**Authors:** Luisa Minghetti, Anita Greco, Maria Puopolo, Marc Combrinck, Donald Warden, A David Smith

**Affiliations:** 1 Department of Cell Biology and Neurosciences, Section of Degenerative and Inflammatory Neurological Diseases, Istituto Superiore di Sanità, Viale Regina Elena, 299, 00161 Rome, Italy; 2 Neurology Unit, Department of Medicine, University of Cape Town, South Africa; 3 The Oxford Project to Investigate Memory and Ageing (OPTIMA), University Department of Pharmacology & Radcliffe Infirmary, Oxford, UK; 4 Oxford Centre for Gene Function, University Department of Physiology, Anatomy & Genetics, Parks Rd, Oxford OX1 3PT, UK

## Abstract

**Background:**

Oxidative stress is believed to be an early event and a key factor in Alzheimer's disease (AD) pathogenesis and progression. In spite of an intensive search for surrogate markers to monitor changes related to oxidative stress in the brain, there is as yet no consensus about which markers to use in clinical studies. The measurement of peripheral anti-oxidants is an alternative way of evaluating the involvement of oxidative stress in the course of the disease. Given the complexity of peripheral anti-oxidant defence, variations in the levels of individual anti-oxidant species may not fully reflect the overall capacity to fight oxidant conditions. We therefore chose to evaluate the total reductive capacity (herein defined as anti-oxidant capacity, AOC) in serum from control subjects and AD patients in order to study the association between peripheral anti-oxidant defence, cognitive impairment and patient survival.

**Methods:**

We measured the levels of AOC in serum samples from 26 cognitively normal controls and 25 AD patients (12 post-mortem confirmed) who completed the Cambridge Cognitive Assessment. Cognitive decline was assessed in a subgroup of 19 patients who underwent a second cognitive assessment 2 years after the initial visit.

**Results:**

Serum AOC levels were lower in AD patients than in controls and were correlated with their cognitive test scores, although AOC levels were unrelated to cognitive decline assessed two years later. On the other hand, AOC levels were predictive of the length of patients' survival, with higher levels giving longer survival.

**Conclusion:**

This study indicates that peripheral anti-oxidant defences are depleted in AD patients. The results suggest that serum AOC is a good index of the general health status and prognosis of patients but does not necessarily reflect the extent to which vulnerable neuronal populations are protected from oxidant processes. Further studies are required to establish whether peripheral AOC measurements may be useful in identifying asymptomatic individuals or those with early symptoms at high risk of developing significant cognitive impairment or dementia.

## Background

Oxidative stress, a condition in which oxidants overwhelm anti-oxidant defences, is associated with ageing and several brain pathologies. In Alzheimer's disease (AD), oxidative stress is one of the earliest events occurring prior to the onset of symptoms and it has been recognised as an essential contributor to the pathogenesis and progression of the disease [1,2].

In the last decade, there has been an intensive search for surrogate markers of brain oxidant injury that could be used to monitor changes related to oxidative stress as well as the efficacy of anti-oxidant therapy. Among the proposed biomarkers are products of lipid peroxidation (e.g. 4-hydroxynonenal and F_2_-isoprostanes), protein oxidation (nitrotyrosine), and DNA oxidation (8-hydroxy-2'-deoxyguanosine, 8-OHdG). However, there is no consensus about which markers can be reliably used in clinical and epidemiological studies, as many of them lack specificity or sensitivity, are subject to the generation of artefacts or are not easily measurable in accessible human samples, such as serum, plasma or urine [3,4].

The measurement of peripheral anti-oxidants is an alternative way of analysing the involvement of oxidative stress in the course of AD and other diseases. Anti-oxidant defences comprise enzymes [superoxide dismutase (SOD) 1 and 2, catalase, glutathione peroxidase (GPx)], as well as many small anti-oxidant molecules, including vitamins A, C and E, carotenoids, glutathione and uric acid. The individual components of peripheral anti-oxidant defence may play similar roles in different cell compartments or they may act in sequence, as in the case of SOD that transforms superoxide into hydrogen peroxide, which is, in turn, broken down into harmless products by catalase or GPx. Some of these components may also be induced by stress, thus increasing the flexibility of the anti-oxidant defence system to react to unfavourable conditions.

Decreases in the levels or activities of anti-oxidant enzymes, such as SOD and GPx, and of both water soluble and lipophilic antioxidant micronutrients such as vitamins A, C and E, and α-carotene, have been reported in AD patients and elderly subjects with mild cognitive impairment [5,6]. In AD plasma levels of anti-oxidants such as lycopene, lutein and α- and β-carotene were inversely correlated with the content of 8-OHdG in lymphocyte DNA [5]. However, the relation between anti-oxidant depletion and clinical outcomes, such as cognitive deficit and patient survival, were not investigated. Epidemiological studies considering dietary or vitamin supplement intake and the risk of dementia have produced conflicting results [7,8]. The evaluation of variations in the levels of individual anti-oxidant species may not fully reflect the overall capacity of the subject to fight oxidant conditions, due to the complexity of peripheral anti-oxidant defences, the induction of compensatory mechanisms and the paradoxical pro-oxidant activity of some anti-oxidants under certain specific conditions.

In an attempt to circumvent the intrinsic problems of measuring single anti-oxidant activities, in the present study we evaluated the total reductive capacity (herein defined as anti-oxidant capacity, AOC) present in serum samples obtained from control subjects and a group of AD patients. The aims of the study were to evaluate the degree to which AD patients are protected from oxidative stress by measuring their peripheral AOC and to examine the association between AOC levels, the severity of cognitive impairment, and patient survival. We found that serum AOC is reduced in AD patients and is associated with their cognitive scores. In addition AOC is predictive of patients' survival but not of cognitive decline assessed two years after AOC determination.

## Methods

### Participants

Participants were volunteers in the Oxford Project to Investigate Memory and Ageing (OPTIMA), a longitudinal observational study established in 1988. OPTIMA protocols have been described previously [9], and were approved by the Central Oxford Research Ethics Committee. The present study concerned 26 cognitively normal controls and 25 patients who satisfied NINCDS-ADRDA criteria for the clinical diagnosis of probable Alzheimer's disease [10]. All participants underwent a physical examination, blood tests and neuroimaging assessments. They also underwent cognitive assessments using the Cambridge Examination for Mental Disorders in the Elderly (CAMDEX) [11], which includes the Cambridge Cognitive Examination (CAMCOG) and the mini-mental state examination (MMSE). The CAMCOG learning subscale (LSS, out of 17) reflects the severity of episodic memory impairment, the cognitive domain that declines first in most patients. The MMSE (out of 30) is a test of global cognitive function that includes questions on orientation in time and space, attention, language, memory and visual construction. The present study excluded volunteers with clinically overt infections, systemic inflammatory conditions, erythrocyte sedimentation rates (ESRs) over 40 mm/hour and those receiving anti-oxidant and or anti-inflammatory treatments. Four participants in each group were sporadic users of low dose aspirin. A subgroup of 19 patients underwent a second cognitive assessment 2.0 (0.2) [median (IQR/2)] years after the initial visit. Of the 13 patients who came to autopsy, 12 were Consortium to Establish a Registry for Alzheimer's disease (CERAD) "definite" and one was CERAD "probable" AD; 2 had Braak limbic stage and 9 had neocortical disease. Of the 3 controls who came to autopsy, 2 were confirmed as CERAD negative; 1 had cortical neuritic plaques.

### Serum collection and AOC assay

Blood was collected in a plain tube without anti-coagulants and left at room temperature for one hour to allow the clot to retract. The sample was then centrifuged at 1000 xg for 5 minutes, at 4°C. The serum supernatant was removed and re-spun for 10 minutes at 1000 g and 4°C. The final supernatant was stored at -70°C prior to assay. The quantification of total reductive capacity in serum samples was determined using an assay kit (P.A.O., MED.DIA, San Germano Vercellese, Italy), which evaluates the reduction of Cu++ to Cu+ by the activity of all anti-oxidants present in the sample [12]. The reduced copper (Cu+) forms a stable complex with bathocuproine that shows an absorption maximum at 490 nm. Values obtained for serum samples were compared with a standard curve of uric acid, used as typical reducing agent. Serial dilutions of each serum sample were analysed in duplicate. Data were expressed as μmoles/L of reducing power. The value of reductive capacity is obtained by multiplying the equivalent in concentration of uric acid by a coefficient that takes into account the oxidation potential of the couple Cu^++^/Cu^+^. The assay was found to be linear from 1 to 1000 μM of uric acid (r = 0.99, P < 0.001). Sensitivity was 22 μM of reductive capacity. Both intra- and inter assay variability showed a coefficient of variance CV that was lower than 4%. Storage time of serum samples did not relate to serum AOC levels (r_s _= 0.20, not significant). In the present study, we used serum samples to avoid any possible interference of anticoagulants with the Cu++ to Cu+ reduction. However, in a pilot study involving 10 subjects, AOC levels in serum and EDTA-plasma in the same subject were significantly correlated (r_s _= 0.772, p = 0.008).

### Apolipoprotein E genotyping

DNA was isolated by standard procedures and the method of Wenham *et al*. [13] was used for Apolipoprotein E (APOE) genotyping.

### Statistical analysis

Non-parametric analysis of variance (Mann-Whitney U-test) was applied to evaluate the differences in AOC concentrations between groups. Data are expressed as median and half the interquartile range (IQR/2). Correlation between experimental variables was measured by the Spearman rank correlation (r_s_). Differences between groups in categorical variables were tested by the chi square test or Fisher's exact probability test. AD patients (n = 25) were split into two subgroups, above and below the observed AOC median value. The Kaplan-Meier method was used to evaluate patient survival for different AOC levels. Statistical inferences were made using the generalised Wilcoxon statistic. A Cox proportional hazards regression model (CPHR) was then used to determine the relationship between survival time and AOC levels, accounting simultaneously for demographic characteristics (sex and age) and MMSE scores. A stepwise procedure was carried out and the relative hazard of dying (RH) and 95% confidence interval (C.I.) were calculated.

## Results

Table 1 shows the demographic and clinical characteristics, cognitive scores and AOC levels of the participants. As expected, we found significant differences between controls and patients with respect to MMSE, LSS and the presence of the APOE ε4 allele. Serum albumin concentrations and ESRs were comparable in controls and patients. The levels of total anti-oxidant defence measured by AOC assay in serum samples were significantly lower in AD patients than in controls (Mann-Whitney U-test, p = 0.0098). Serum AOC levels were not related to the age of the subjects.

**Table 1:** Demographic and clinical characteristics of controls and patients

	Controls	Patients	p
Total number of subjects	26	25	
Sex	15F/11M	17F/8M	ns
Age (years)	71.1 (6.4)	71.1 (6.0)	ns
MMSE	28.0 (0.5)	21.0 (5.0)	< 0.0001*
LSS	14.0 (1.0)	4.0 (2.5)	< 0.0001*
APOE ε4 (%)	23	68	0.0019**
Serum albumin (g/L)	43 (1.5)	44 (2.5)	ns
ESR (mm/hour)	9.5 (5.5)	9.0 (5.0)	ns
AOC (μmoles/L)	1197.5 (91.9)	1103.0 (99.5)	0.0098*

Data are expressed as median (IQR/2); ns = not significant; * Mann-Whitney *U *test, ** Fisher's exact probability test

**Figure 1:**
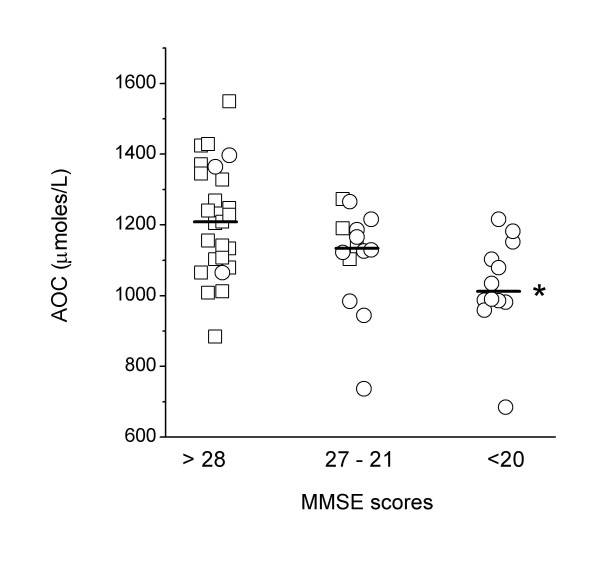
Serum AOC levels in participants grouped according to MMSE scores. Square and circle symbols indicate controls and Alzheimer's disease patients, respectively. *p = 0.0018 versus >28 MMSE score group.

To investigate the possible relationship between serum AOC and cognitive deficit, we used the MMSE scores as proxy of status (control vs AD) and of disease progression. Subjects were divided into three mutually exclusive groups ranked by increasing MMSE scores [below 20 (n = 12)], between 21 and 27 (n = 14), and above 28 (n = 25)]. The three groups were homogeneous with respect to age [70.8 (5.4), 70.7 (6.8) and 72.0 (7.3) for low, intermediate and high MMSE scores, respectively], but differed regarding the presence of at least one APOE ε4 allele (83, 57 and 20% for low, intermediate and high MMSE scores, respectively). As shown in Figure 1, there was a tendency towards AOC levels being positively associated with cognitive performance: the median AOC value of the group with the lowest MMSE score range was significantly lower than that of the group with the highest MMSE scores [1012.5 (78.4) and 1209.0 (131.7) μmoles/L, respectively; p = 0.0018]. The middle range group (MMSE scores between 21 and 27) showed an intermediate AOC level, which did not differ from the levels of the two extreme groups. Similar results were obtained when the analysis was carried out using LSS scores as proxy of status (p < 0.025, lowest vs highest LSS scores).

**Figure 2:**
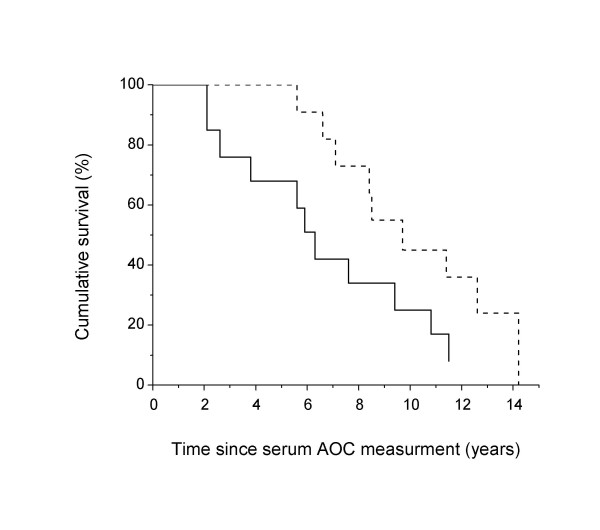
Cumulative survival of patients with Alzheimer's disease with low (solid line) and high (dashed line) initial AOC levels. Low and high AOC levels were defined as below or above the median AOC levels of the patient group (1103.0 μmoles/L). Cox proportional hazards regression.

As shown in Figure 2, high serum AOC levels predicted a longer survival of AD patients (CPHR, co-varying MMSE scores, age and sex). To illustrate this, AD patients were divided into two groups according to their serum AOC levels, below or above the median value of 1103.0 μmoles/L. The median residual survival of patients (years of survival since AOC measurement) was 5.9 years for the subgroup with low AOC levels and 9.1 years for the subgroup with high AOC level (generalized Wilcoxon test p = 0.032). In the Cox proportional hazards model, the stepwise procedure accounting simultaneously for demographic characteristics (sex and age) and MMSE scores, selected only the AOC level as a factor significantly associated with survival of patients. The hazard of dying was lower in patients with elevated AOC levels than in patients with low AOC levels (RH = 0.34; 95% C.I. = 0.12–0.97). The frequency of subjects with at least one APOE ε4 allele was not different in the two groups with low or high AOC levels (69.2 and 66.6%, respectively).

**Figure 3:**
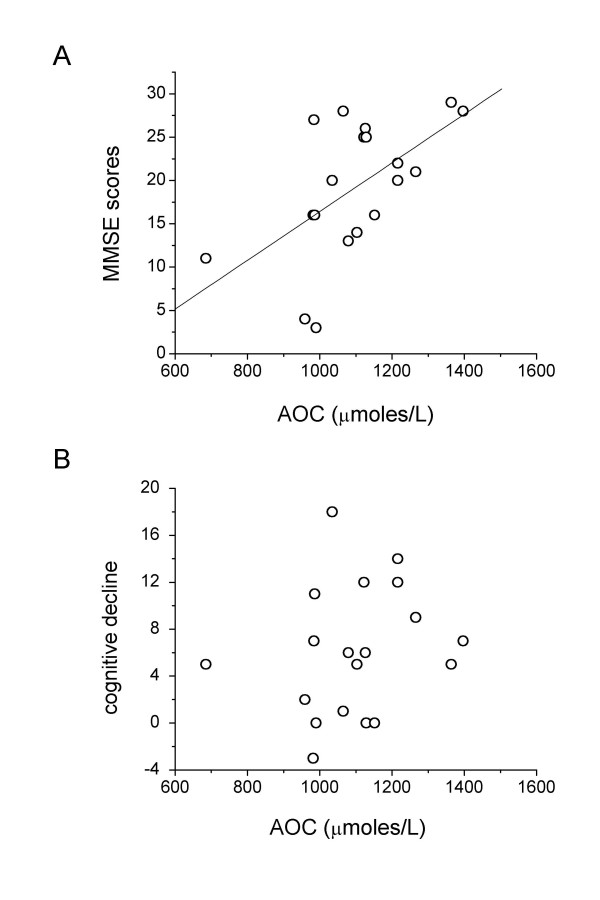
Dependence of serum AOC levels on MMSE scores (A) or on cognitive decline (B), defined as the difference between MMSE scores over a period of 2.0 (0.2) [median(IQR/2)] years.

Since a subgroup of patients (n = 19; 13F/6M) underwent a second cognitive assessment 2.0 (0.2) years after the first visit, we analysed whether serum AOC level could also predict the degree of cognitive deficit two years later. The characteristics of the subgroup at first visit [median age 71.1 (6.4); APOE ε4 68.4%; MMSE 20.0 (5.3), LSS 4.0 (3.5) and AOC 1103.0 (98.0) μmole/L] were not different from those of the whole AD group. As shown in Figure 3, serum AOC levels correlated with the MMSE scores at the time of blood sampling (Fig. 3A, r_s _= 0.567, p = 0.011, n = 19), but not with the cognitive decline over the 2 years of observation, calculated as difference between MMSE scores obtained at the two visits (Fig. 3B, r_s _= 0.275, p = 0.253, n = 19). A similar picture was observed for LSS scores: AOC levels were correlated with LSS scores (r_s _= 0.540, p = 0.016, n = 19) at initial assessment but not with the cognitive decline (r_s _= 0.003, p = 0.991).

## Discussion

This study shows that the peripheral anti-oxidant defence system in AD patients is significantly impaired. The level of anti-oxidant defence is predictive of patients' survival but not the cognitive decline over the subsequent two years.

The lower levels of AOC in AD patients is consistent with the lower levels and/or activities of individual components of the peripheral anti-oxidant defence system previously reported in subjects with AD or mild cognitive impairment [5,6]. However, other studies failed to confirm the findings of depletion of specific plasma anti-oxidants such as vitamins A and E [8]. Variations in individual anti-oxidant activities *per se *may not, in fact, be very relevant. In a population based study, Berr et al., [14] showed that the cognitive decline in subjects whose MMSE scores dropped by at least three points after a four year follow-up was accompanied by decreased levels of selenium-dependent GPx activity but increased levels of Cu/Zn-SOD activity. The association of the enzymatic activities with cognitive decline was no longer significant when adjusting for MMSE, age, sex, education and other factors related to life style. However, the ratio Cu/Zn-Sod / GPx, which was about 10% higher in subjects with cognitive decline, remained significantly associated with cognitive decline after adjustment.

The evaluation of the total reducing capacity of biological fluids such as serum or plasma may provide a better estimation of the peripheral resistance to oxidant injury than the measurement of a set of individual anti-oxidant species. The anti-oxidant activity measured by the AOC assay is the net result of the contribution of all the individual anti-oxidants, of their interactions and complexities. Total plasma anti-oxidant activity has been measured previously, although different methods have been used [15-17]. One of these methods, measuring the hydrosoluble anti-oxidant status of biological fluids such as plasma, led to conflicting results [15,16]. In a recent study using a spectrophotometric method, total anti-oxidant activity was decreased in AD patients and showed a tendency towards a slight negative correlation with clinical duration as defined by the time between first symptoms and clinical diagnosis [17].

To our knowledge, our study is the first one attempting to correlate peripheral anti-oxidant defence with the progression of cognitive deficit in a well defined group of AD patients. Although AOC levels were positively associated with MMSE and LSS scores at the time of blood sampling for AOC determination, they did not predict the cognitive deficit two years later. Although our study may have been underpowered to detect such an association, our findings suggests that the level of peripheral anti-oxidant defence may be indicative of peripheral oxidant status rather than of central processes related to neurodegeneration and cognitive decline. However, we cannot rule out the possibility that the rate of cognitive decline might be associated with AOC levels at later time points. Longitudinal studies over extended periods of time (more than 2 years) should help to clarify this issue.

Another important and novel observation of our study is the positive relationship between peripheral reductive capacity and survival of AD subjects. According to the Cox proportional hazards model, the hazard of dying was lower in patients with elevated serum AOC levels (above the median level of 1103.0 μmoles/L) than in patients with low AOC levels. The median residual survival of patients with high serum AOC was about three years longer than that of subjects in the subgroup with low AOC levels. Since participants taking anti-inflammatory drugs and/or anti-oxidant supplements were excluded from the study, these findings suggest that AOC might be a good indicator of the general health status of the body and of its capacity to cope with inflammatory and oxidative insults.

The lower levels of peripheral AOC observed in AD patients is also consistent with the finding of lower oxidative resistance of plasma and CSF from AD patients compared with controls [18]. These findings further indicate that oxidative stress is a pervasive condition, which not only affects selectively vulnerable neuronal populations but also occurs in the periphery, as indicated by the higher 8-OHdG content in the lymphocyte DNA of AD patients compared with controls [5].

## Conclusion

This study indicates that the levels of peripheral anti-oxidant defence, measured by an assay that evaluates the total reductive capacity present in serum, are decreased in AD and are positively associated with the survival of patients. The AOC levels were associated with cognitive scores, but they were not predictive of the progression of cognitive deficit. Serum AOC may therefore be a good index of the general anti-oxidant status of the body but does not necessarily reflect the body's capacity to protect vulnerable neuronal populations from oxidant processes. Our study does not, however, rule out the possibility that serum AOC measurements may be useful in identifying asymptomatic subjects or those with early cognitive symptoms who are at risk of progressing to more severe cognitive impairment or dementia.

## Competing interests

The author(s) declare that they have no competing interests.

## Authors' contributions

LM participated in the design of the study, performed AOC assay and prepared the manuscript; AG performed AOC assay and help to draft the manuscript; MP performed statistical analysis and help to draft the manuscript; DW participated in the design of the study, was involved in collection of blood samples and performed ApoE genotyping; MC participated in the design of the study, was involved in clinical examinations and help to draft the manuscript; ADS participated in the design of the study and helped to draft the manuscript.
